# Development of an HPLC-DAD Method for the Quantification of Ten Compounds from *Moringa oleifera* Lam. and Its Application in Quality Control of Commercial Products

**DOI:** 10.3390/molecules25194451

**Published:** 2020-09-28

**Authors:** Ramakwala Christinah Chokwe, Simiso Dube, Mathew Muzi Nindi

**Affiliations:** Department of Chemistry, The Science Campus, College of Science Engineering and Technology, University of South Africa, Corner Christiaan de Wet and Pioneer Avenue, Florida Park, Roodepoort 1709, South Africa; christinah.chokwe@gmail.com (R.C.C.); dubes@unisa.ac.za (S.D.)

**Keywords:** method development, HPLC-DAD, *Moringa oleifera*, quality control

## Abstract

An HPLC-DAD separation method for the simultaneous quantification of ten compounds from *Moringa oleifera* plant was developed. The method was validated with pure solvent and different matrices of *M. oleifera* products. This method was found to be linear in the concentration range of 1 to 10 mg L^−1^ for all the compounds in the solvent and from 3 to 10 mg kg^−1^ in the different matrices. The correlation coefficients ranged between 0.9900 and 0.9999. Intra-day and inter-day variability showed that the developed method is both repeatable and precise with percent relative standard deviation values less than 10% and 20%, respectively. Limits of detection ranged between 0.06 and 0.8 mg L^−1^ for the solvent and 0.1–1.5 mg kg^−1^ for the matrices, while the limit of quantification ranged between 0.2 and 2.8 mg L^−1^ and 0.4–4.8 mg kg^−1^, respectively. The validated method was applied successfully to thirty-two different *M. oleifera* products, whereby all ten compounds were detected in one of the samples. Principal component analysis was used to assess the correlation and variance between the products. Variations were observed in products from different regions and from different manufacturers.

## 1. Introduction

*Moringa oleifera* Lam. is the most cultivated species in the Moringa genus. It is a valued natural product because of its many uses and hence it has earned names such as the miracle tree and the magic bullet. All parts of the plant are useful in some way or another, either medical, nutritional, or cosmetic [1]. The plant is used traditionally to treat cholera, diabetes, skin infections, respiratory problems, and ear infection, among others [1,2]. The seeds of the plant are also used to purify water, as demonstrated in studies reported by Ndabigengesere et al., Sanchez-Martin et al. and Suhartini et al. [3,4,5]. Its popularity has led to commercialization of products prepared from the plant.

There are efforts in emerging economies to use natural products as alternatives to synthetic drugs that are usually associated with side effects and are expensive. Growing interest in research on these products observed lately attests to this. Phytochemical studies support traditional use of the plant and validate the commercialization of *M. oleifera* products [6,7,8]. The phytochemical approach is generally used to evaluate the activity of the plant extracts, and thereafter, the active compounds are isolated and identified. However, sometimes the activity of plant extracts has been reported to be greater than that of the individual compounds [9]. This is probably due to the synergistic effect of the compounds within the extract, as shown in studies conducted by Brahmbhatt et al. [10]. Most commercial natural products available in the market are sold as whole extracts for this reason. A knowledge gap has been identified in terms of quality control and quality assurance measures for various natural products in the market. Quality control is important to avoid low-quality products being delivered to consumers as well as to prevent potential harmful side effects. It is therefore important to develop analytical methods for quality control of medicinal plant products. Shanker et al. developed a quantification method for two nitrile glycosides in the leaves and bark of *M. oleifera* [11]. Similarly, Pachauri et al. developed a quantification method for 1,3-dibenzyl urea and aurantiamide acetate found in the roots *of M. oleifera* [12].

**Figure 1:**
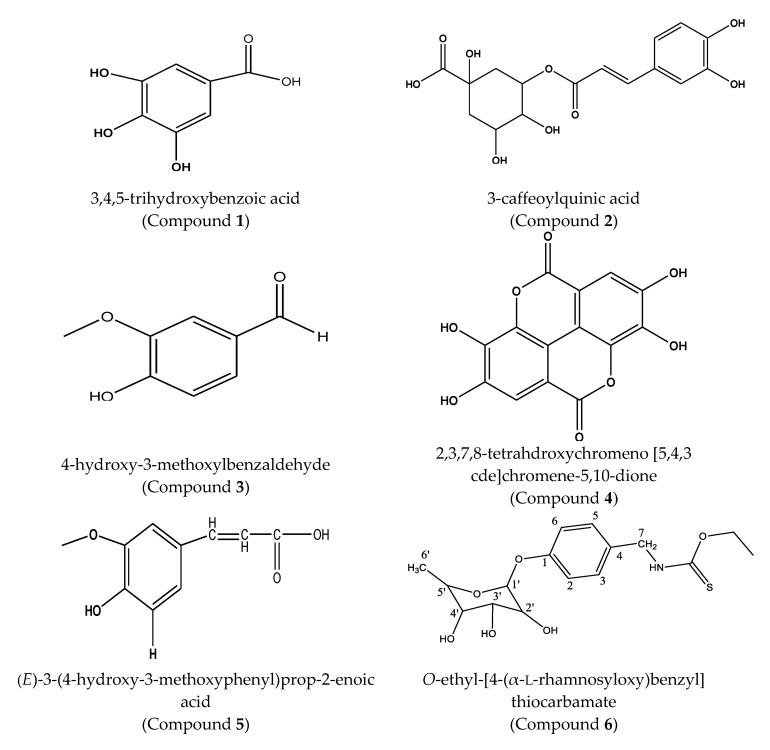

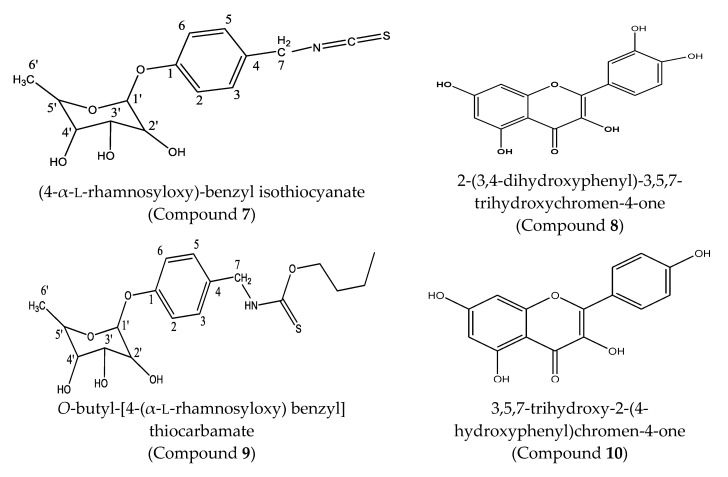
Structures of the analyzed compounds in *Moringa oleifera* Lam.

The purpose of this study was to develop and validate a simple method for the simultaneous separation and quantification of ten compounds that are commonly found in different parts of *M. oleifera* plant (illustrated in Figure 1) in order to perform their analysis in different *M. oleifera* plant and commercial products (*n* = 32) [13,14,15,16]. HPLC-DAD was the selected method because it fulfils the purpose. Small production entities and/or companies could use this method to assess the quality of their products, which is an efficient and low-cost method with adequate sensitivity for the studied metabolites. In addition, the sensitivity offered by an HPLC-DAD is adequate for most compounds found in plants.

To the best of our knowledge, no work has been reported on simultaneous quantitative determination of the ten compounds in *M. oleifera* selected for this study.

## 2. Results

### 2.1. HPLC-DAD Method Development for Separation and Quantification

**Figure 2:**
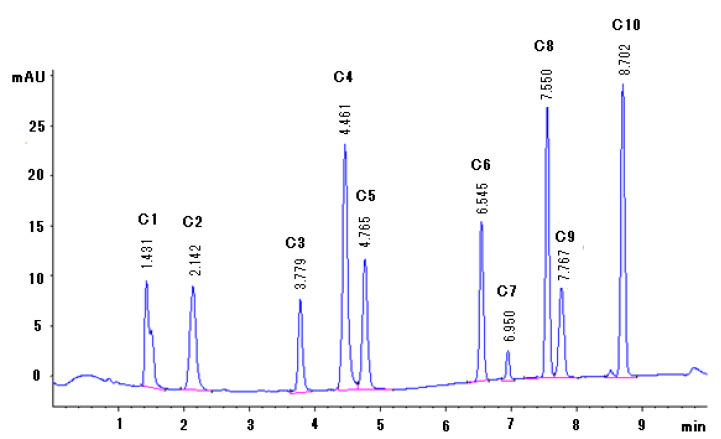
Chromatogram representing the separation of Compounds **1**–**10** using an XTerra^®^ MS C18 column (150 × 4.6 mm, 3.5 µm) under the conditions described in Section 3.3.

Compounds **1**–**10**, illustrated in Figure 1, were used as reference standards to develop an HPLC-DAD separation method that could be used for qualitative and quantitative analysis of the *Moringa oleifera* products. Figure 2 shows a typical chromatogram of a baseline separation of the ten compounds eluting in the following order with retention times (t_R_) as given: compound **1** (t_R_: 1.4 min), **2** (t_R_: 2.1 min), **3** (t_R_: 3.8 min), **4** (t_R_: 4.5 min), **5** (t_R_: 4.8 min), **6** (t_R_: 6.5 min), **7** (t_R_: 7.0 min), **8** (t_R_: 7.6 min), **9** (t_R_: 7.8 min), and **10** (t_R_: 8.7 min), under the conditions described in Section 3.3. Baseline separation for all the compounds was achieved and allowed accurate quantification of the compounds.

### 2.2. Validation of the HPLC-DAD Method

#### 2.2.1. Linearity and Matrix Effect

**Figure 3:**
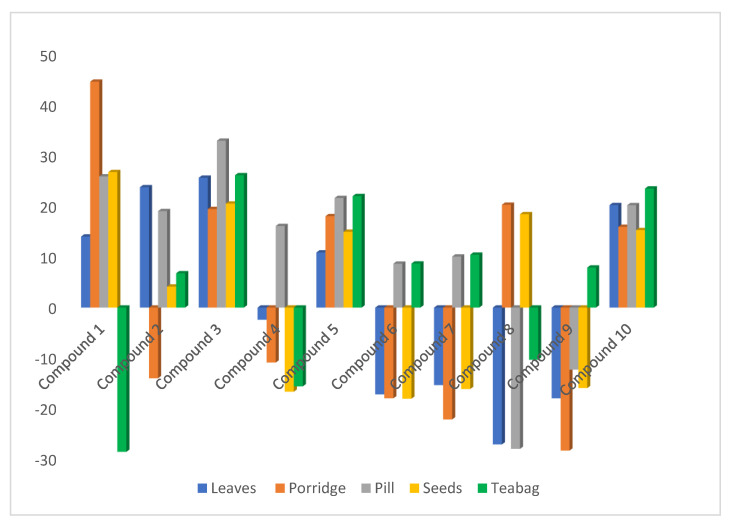
Representation of the percentage matrix effect experienced by the compounds in the different matrices.

Linearity of the method was determined using the external standard calibration and matrix-match calibration methods. The calibration curves were obtained by plotting the average peak area vs. the concentration of the compounds. The calibration curves for all the compounds in the solvent and five different matrices showed a good linear relationship between peak area and concentration in the range of 1–10 mg L^−1^ and 3–10 mg kg^−1^, respectively. This is shown by the regression coefficients (r^2^) of the compounds, that were in the range 0.9942–0.9999 and 0.9900–0.9998 in the solvent and matrices respectively, which confirms good linearity (Table 1). The percentage matrix effect for each compound in the different matrices were calculated using Equation (1) given in Section 3.4. As shown in Figure 3, the compounds experience either a positive or negative matrix effect. A negative matrix effect means that the detector response for the compound was decreased/suppressed, while a positive matrix effect means that the detector response was increased/enhanced [17]. The % matrix effect (ME) for the compounds in the different matrices ranged from −29% to 45%, with compound **1** experiencing the highest suppression in the teabags and the highest enhancement in the porridge sample as compared to the other compounds. Compounds **3**, **5**, and **10** were enhanced in all the products shown by the positive matrix effect. Overall, 60% of the compounds were enhanced and 40% were suppressed in the different products, as shown by the positive and negative matrix effects, respectively. Matrix effect is classified as mild, medium, or strong when % ME is <±20%, ≥±20% but <±50% and ≥±50%, respectively. In the leaves, porridge, and teabags matrices, 60% of the compounds experienced mild matrix effect while 40% experienced medium matrix effect (see Supplementary Figure S1). With regards to the seeds, 80% and 20% of the compounds experienced mild and medium matrix effect, respectively (Supplementary Figure S1). On the other hand, for the pill matrix, there was equal distribution of mild and medium matrix effects (Supplementary Figure S1). Strong matrix effect was not observed for any compound in all the products. This means that the matrix effect experienced by the compounds in the different products is not significant and hence calibration equations in the solvent can be used for quantification. However, matrix-matched calibration equations were used to give more accurate results.

Both limit of detection (LOD) and limit of quantification (LOQ) were determined using the linear regression equation according to the ISO11843 method, where LOD is signal-to-noise ratio (S/N) × 3, and LOQ is signal-noise-ratio (S/N) × 10. Table 1 shows LODs and LOQs in the range 0.1–0.8 mg L^−1^ in the solvent, while LOQs were in the range 0.2–2.8 mg L^−1^. For the leaves, the LODs ranged from 0.5 to 1.4 mg kg^−1^, while LOQs were 1.5–4.8 mg kg^−1^. For the remaining products (i.e., porridge, pill, seeds, and teabag), the LODs and LOQs ranged from 0.1 to 1.2 mg kg^−1^ and 0.4 to 4.6 mg kg^−1^, respectively.

#### 2.2.2. Recovery, Precision, and Uncertainty Measurements

The percentage recoveries for the compounds at the lowest spiking concentration (3 mg kg^−1^) for the compounds in the leaves, porridge, pill, seeds, and teabags ranged between 75.7 and 113.0, 75.9 and 100.6, 72.0 and 100.2, 66.7 and 108.9, and 69.7 and 99.2, respectively. At the medium spiking concentration (6 mg kg^−1^), the percentage recoveries for the compounds ranged between 84.8 and 104.7, 87.5 and 101.6, 90.5 and 101.3, 84.2 and 105.4, and 91.0 and 97.8 for the leaves, porridge, pill, seeds, and teabags, respectively. The percentage recoveries at the highest spiking concentration (10 mg kg^−1^), were 91.1–100.5, 98.9–102.0, 98.2–101.0, 99.0–102.3, and 98.2–103.6 for the leaves, porridge, pill, seeds, and teabags respectively, as shown in Table 2. The recoveries in different matrices had acceptable random errors, as indicated by the percent relative standard deviation (%RSD) values which were below the 20% threshold as stipulated by the EU Commission Decision 2002/657/EC directive [18]. These results indicate that the method is accurate.

Intra-day and inter-day variability were determined using the lowest, middle, and highest concentrations. The results were expressed as percent relative standard deviation (%RSD). Table 2 shows intra-day results, where %RSD values for all the compounds in the solvent were found to be in the range of 0.3–1.1%, 0.2–0.8%, and 0.2–0.4% at the lowest, medium, and highest concentrations. The %RSD values for the compounds in the different matrices were 0.2–4.7, 0.2–3.2, and 0.1–1.4 at the lowest, medium, and highest concentrations, respectively. Inter-day variability was used to assess reproducibility over five consecutive days. The %RSD values (Table 2) obtained for the solvent ranged between 0.2% and 1.6%, 0.05% and 0.6%, and 0.02% and 0.2% for the lowest, medium, and highest concentrations. In the different matrices, the %RSD values for the compounds ranged between 0.3 and 6.3, 0.1 and 4.4, and 0.2 and 4.6 for the lowest, medium, and highest concentrations. The values obtained for intra-day and inter-day variability were below 10% and 20% respectively, which is considered acceptable and indicates that the method is both repeatable and reproducible according to the EU Commission Decision 2002/657/EC directive [18].

The measurement uncertainty due to precision and recoveries were calculated using Equations (3)–(5), the results are given in Table 2. The results are reported at the 95% confidence level. The measurement uncertainty ranged from 0.3% to 9.8% with compound **2** exhibiting the highest uncertainty in the teabag matrix. This is because of the low recovery of the compound at the lowest concentration level. Compound **3** showed the lowest uncertainty in the leaves which is due to the good recovery and %RSD of the compound in the matrix.

#### 2.2.3. Specificity

Specificity of the method was determined using the DAD Chemstation software to assess the purity of the compounds of interest in the different matrices. Their Ultraviolet (UV) spectra and retention times were also compared with those in the pure solvent. The purity indices were in the range of 897 to 998 for all the compounds in the different matrices, meaning that they were not co-eluting. The UV spectra and retention times of all the peaks corresponding to the compounds of interest in the different matrices were similar to those in the solvent with standard deviations between 0.02 and 0.14. This showed that the method was specific for the compounds of interest.

### 2.3. Quantification of the Compounds in the Plant and Products’ Extracts

**Figure 4:**
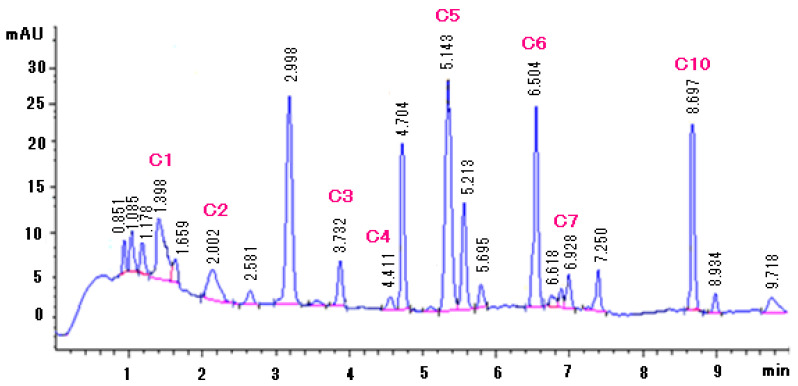
Chromatogram representing the separation of leaves L**4** sample using the developed and validated method.

The performance of the developed and validated method was tested on *M. oleifera* products which included the pills (P), porridge (PRD), teabags (TB), leaves (L), and seeds (S) from different regions (Table 3). Table S2 shows the information about when and where the leaves and seeds samples were collected. Retention times and UV spectra of the identified compounds were compared with those of the reference standards (See Figure 4 below for a chromatogram representing the sample L**4** and Supplementary Figures S3–S8 for chromatograms of the standards and selected samples plus UV spectra of the peaks of interest). The matrix-matched linear regression equations were used to calculate the concentrations of the compounds in the different products. It is noted that even though all the compounds in this study have been reported previously in the seeds and leaves (except for compound **9** which has only been reported in the seeds), only compounds **6** and **7**, known for their anticancer properties, were detected in all the samples. Compound **6** was present in higher amounts in leaves L**3** from Limpopo, South Africa (9.7 mg kg^−1^), while the PRD**1** from Manufacturer I contained the highest amount of compound **7** (9.6 mg kg^−1^). As much as compounds **6** and **7** were detected in all the samples, they could not be quantified in S**6**, PRD**3** and PRD**4** samples because they were present in quantities below LOQ. L**4** from Mpumalanga contained all the ten compounds, however, five out of the ten were below the LOQ. L**5**, TB**1**, TB**2**, and TB**3** samples contained five compounds each, corresponding to the least number among all 32 samples. There are some prominent peaks which were observed in the matrices but could not be identified due to insufficient data. These prominent peaks were observed at retention times of 1.2 to 1.4 min in the samples L**4**, P**4**, and PRD**1**. Other prominent peaks were observed at 5.2 to 5.8 min in L**4**, P**4**, S**2**, PRD**1**, and TB**7** (See Supplementary Figures S4–S8).

**Figure 5:**
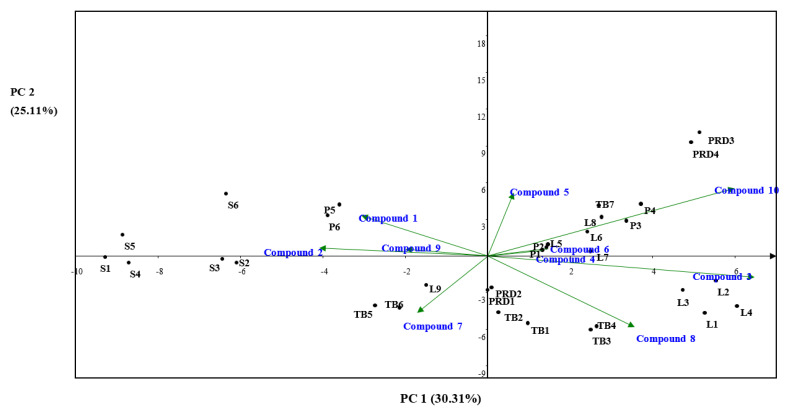
Principal component analysis (PCA) biplot for thirty-two *M. oleifera* samples.

The variance and correlation between the different products were assessed using rincipal component analysis (PCA) and hierarchical clustering. Figure 5 shows a PCA biplot of the products and the compounds. The plot shows that PC1 explains 30.3% of the variance while PC2 explains 25.1%. The leaves in general apart from L**9** are negatively correlated to the seeds along the PC1 axis. This is also observed on the hierarchy clustering plot where groups 2 and 4 containing these samples are correlated at a lower level (Supplementary Figure S2). The leaves from Mpumalanga are clustered together because they all contain significant amounts of compound **3**. The same is true for PRD**3** and PRD**4** which contain compound **10**. PC2 shows the difference between the leaves from Limpopo and those from Mpumalanga, though the variation is not significant. The significant difference between the leaves from Ethiopia and those from South Africa is shown by their separation along PC 1. This difference could be due to geographical effects. The variation between manufacturers of the same products was also assessed by the PCA plot. The plot shows that even though products from the same manufacturer are closely correlated, there is a significant variation between products from different manufacturers. For example, P**1** and P**2** (from M**I**) are negatively correlated to P**5** and P**6** (from M**III**) along PC1, which is also confirmed by the hierarchical cluster plot (Supplementary Figure S2) where these are correlated at a lower level (groups 3 and 4). These results prove the need for quality control methods for the products in the market.

## 3. Materials and Methods

All the solvents used in this work were of HPLC grade with a purity > 99.9%. Some of the reference standards (Compounds **6**, **7**, and **9**) were isolated in our laboratory (see Supplementary Table S1) with a percentage purity ranging from 90 to 99 [19]. The rest were purchased from Sigma-Aldrich (Steinheim, Germany) and had percentage purity between 95 and 97. The commercial *M. oleifera* products used as samples such as the pills, porridge, and teabags were purchased from local pharmacies. The leaves and seeds were collected from different regions (see Table S2) and were authenticated by the College of Agriculture and Environmental Sciences at the University of South Africa. The aqueous solutions were prepared using ultra-high-purity (UHP) water (18.2 mΩ) from a Milli-Q water purification system (Molsheim, France) and filtered using a 0.22 µm membrane filter from Sigma-Aldrich (Steinheim, Germany).

### 3.1. Instrumentation

The compounds were extracted using a Microwave reaction system: Multiware 500 (Anton Paar, Graz, Austria). The HPLC method for separation and quantification of the compounds was developed using an Agilent HPLC 1260 system (Agilent Technologies, Waldbronn, Germany) which consisted of a binary high-pressure pump, autosampler, a thermostatted column compartment, a diode array detector, and a fluorescence detector. Instrument control, data collection, and processing were achieved using the Chemstation (version 1.9.0) software.

### 3.2. Extraction Procedure

The leaves and seeds were grinded using mortar and pestle to give fine powders. Two grams of each sample were extracted using an optimized microwave-assisted extraction method for phenolic compounds in *M. oleifera* reported by Rodriguez-Pérez et al. [20]. The extraction was performed for each sample using 20 mL of water:ethanol (58:42 *v*/*v*) at a temperature of 158 °C for 20 min. The extracts were then filtered using Whatman filter paper and dried using a freeze-drier. The dried extracts were stored at 4 °C until further use.

### 3.3. Chromatographic Procedure for Quantification of the Compounds

The stock solutions were prepared using the isolated and commercial standards. These were dissolved in an acetonitrile:water solution (1:1, *v/v*). The synthetic mixture of the ten compounds was prepared using the stock solutions. The separation of the mixture was performed on an XTerra^®^ MS C18 (150 × 4.6 mm, 3.5 µm) analytical column. The mobile phase used for the separation was acetonitrile and 0.1% formic acid in water. The optimum mobile phase composition was as follows: a gradient elution mode commencing at 0 min, 80% (water), 2 min, 75% (water), 3 min, 70% (water), 4 min, 65% (water), 5 min, 55% (water), 6 min, 50% (water), 7 min, 45% (water), and 8 min, 40% (water), with an elution time of nine minutes and post time of one minute. The optimum flow rate and temperature were 1.3 mL min^−1^ and 35 °C, respectively. The injection volume was 5 µL and the separation was monitored at 254 nm. The compounds elute in the following order: compound **1** (t_R_: 1.4 min), **2** (t_R_: 2.1 min), **3** (t_R_: 3.8 min), **4** (t_R_: 4.5 min), **5** (t_R_: 4.8 min), **6** (t_R_: 6.5 min), **7** (t_R_: 7.0 min), **8** (t_R_: 7.6 min), **9** (t_R_: 7.8 min), and **10** (t_R_: 8.7 min).

### 3.4. Method Validation

The developed method was validated using the following parameters: linearity, method limit of detection (LOD), method limit of quantification (LOQ), accuracy, precision, measurement uncertainty, and specificity. The calibration standard solutions in the solvent were prepared by serial dilution using the stock solutions as described in Section 3.3. A seven-point calibration curve was plotted covering concentration ranges of 1 to 10 mg L^−1^ for compounds with seven replicates at each concentration level. The different products were used separately for the matrix-match calibration. The matrix-match standards were prepared by spiking appropriate amounts between 3 to 10 mg kg^−1^ of the stock solutions into homogenized mixtures of the products and extracted using the method described in Section 3.2. A five-point calibration curve (3–10 mg kg^−1^) was used for the matrix-match calibration with seven replicates. The slopes of the curves for both methods were used to calculate the percentage matrix effect (%ME) for each compound using the equation:

% ME = (S _matrix_ − S _solvent_/S _solvent_) × 100%. (1)

The recoveries were determined at three concentration levels, 3, 6, and 10 mg kg^−1^, with seven replicates at each concentration level. The samples were spiked with appropriate amounts and extracted using the method described in Section 3.2. The % recoveries for the compounds were calculated using the equation:

% Recovery = (C _found_ − C _original_/C _spiked_) × 100% (2)

Intraday variability for the compounds in the solvent and the different matrices was assessed in one day at the lowest, medium, and highest concentrations. Intermediate precision was determined as described for the intra-day variability, however, this was done over five consecutive days.

The measurement uncertainty was estimated according to the top-down approach. The expanded uncertainty was obtained at the 95% confidence level using the lowest and highest concentration levels. Precision and recoveries were the major source in the uncertainty budget. Measurement uncertainty (U) due to precision of recoveries was calculated using the following equation [17]:

(3) $$\mathrm{UX}\;(\%)\;=\;\sqrt{RSD^{2}+{\left(\frac{100\;-\;R}{\sqrt[{2}]{3}}\right)}^{2}}.$$

where RSD is the percentage relative standard deviation and R is the percentage recovery. The combined measurement uncertainty was calculated using the equation:

(4) $$\mathrm{Uc}\;(\%)\;=\;\sqrt{{\left(U_{3}\right)}^{2}+{\left(U_{10}\right)}^{2}}$$

where *U*_3_ is the uncertainty at 3 mg kg^−1^ and *U*_10_ is the uncertainty at 10 mg kg^−1^. The expanded measurement uncertainty was calculated using the equation:

U (%) = U_C_ × K (5)

where K is the coverage factor 2 at the 95% confidence level.

### 3.5. Quantification of the Compounds in the Real Samples

The method was applied to *M. oleifera* leaves, seeds, and products purchased commercially. The commercial samples corresponded to *M. oleifera* leaves, expect the seed samples. All the samples were extracted using the method described in Section 3.2. The extracts of the different products were dissolved individually in acetonitrile and water (1:1, *v*/*v*) and then filtered using 0.22 µm Polyvinylide fluoride (PVDF) syringe filters. The samples were analyzed using the validated method. PA leontological STatistics (PAST) version 217 software was used to assess the correlation and variance between the products. 

## 4. Conclusions

A short and precise method was developed and validated using HPLC-DAD for the simultaneous quantification of ten compounds found in *Moringa oleifera* plant. The method was successfully applied to 32 different samples comprising plant materials, including leaves (*n* = 9), seeds (*n* = 6), and commercial products (*n* = 17). The two anticancer compounds, i.e., 4(α-l-rhamnosyloxy)-benzyl isothiocyanate and *O*-ethyl-(4-(α-l-Rhamnosyloxy) benzyl) thiocarbamate were detected in all the products in the range 3.5–9.7 mg kg^−1^ and <LOQ to 9.6 mg kg^−1^, respectively. 4-hydroxy-3-methoxylbenzaldehyde and 3,5,7-trihydroxy-2-(4-hydroxyphenyl) chromen-4-one, which are reported to have antibacterial, anti-inflammatory, and antioxidant properties, were detected in ten products. This means the products could be beneficial to consumers. The results obtained from PCA and cluster analyses indicate variations between products from different manufacturers and regions. Therefore, it can be concluded that the developed method may be used to assess the quality of various *Moringa oleifera* products.

## Figures and Tables

**Table 1:** Linear regression equations (correlation coefficients) where Y is the response of the compound and x is the concentration of the compound, limits of detection ^#^, and limits of quantification * for the solvent and different matrices.

Compound	Solvent (mg L^−1^)	Leaves (mg kg^−1^)	Porridge (mg kg^−1^)	Pill (mg kg^−1^)	Seeds (mg kg^−1^)	Teabag (mg kg^−1^)
**1**	Y = 5.6426x + 0.1675	Y = 6.4329x + 0.3185	Y = 8.1535x + 8.0563	Y = 7.1004x + 4.761	Y = 7.1488x + 1.322	Y = 4.0215x + 2.1731
	(0.9992), 0.4 ^#^, 1.5 *	(0.9920), 1.4 ^#^, 4.7 *	(0.9926), 1.4 ^#^, 4.5 *	(0.9929), 1.3 ^#^, 4.4 *	(0.9946), 1.4 ^#^, 4.6 *	(0.9914), 0.8 ^#^, 2.6 *
**2**	Y = 5.2976x + 1.7616	Y = 6.5537x + 0.3585	Y = 4.5566x + 0.0311	Y = 6.304x − 0.1218	Y = 5.5164x − 1.0216	Y = 5.655x + 0.0956
	(0.9983), 0.2 ^#^, 0.8 *	(0.9917), 0.9 ^#^,2.8 *	(0.9980), 0.6 ^#^, 2.1 *	(0.9998), 0.4 ^#^, 1.2 *	(0.9977), 0.8 ^#^, 2.5 *	(0.9910), 1.5 ^#^, 2.9 *
**3**	Y = 3.5287x + 0.0056 (0.9997)	Y = 4.4315x − 0.1674	Y = 4.2137x − 0.7968	Y = 4.6886x − 0.0703	Y = 4.2517x − 0.9134	Y = 4.4485x − 1.2286
	0.2 ^#^, 0.6 *	(0.9918), 1.4 ^#^, 4.8 *	(0.9978), 0.7 ^#^, 2.5 *	(0.9998), 0.2 ^#^, 0.8 *	(0.9972), 0.8 ^#^, 2.8 *	(0.9953), 0.9 ^#^, 3.6 *
**4**	Y = 17.023x + 0.2835	Y = 16.614x + 1.8819	Y = 15.178x + 1.3813	Y = 19.759x − 0.7674	Y = 14.183x − 0.8676	Y = 14.365x + 0.2648
	(0.9989), 0.1 ^#^, 0.2 *	(0.9900), 0.5 ^#^, 1.8 *	(0.9944), 0.2 ^#^, 0.8 *	(0.9942), 1.2 ^#^, 4.1 *	(0.9957), 0.3 ^#^, 1.0 *	(0.9928), 0.1 ^#^, 0.4 *
**5**	Y = 6.7695x + 2.3567	Y = 7.5045x + 0.1158	Y = 7.9884x − 1.3053	Y = 8.2307x + 0.2162	Y = 7.7802x − 0.1998	Y = 8.256x − 2.4614
	(0.9998), 0.2 ^#^, 0.6 *	(0.9930), 0.5 ^#^, 1.5 *	(0.9983), 0.6 ^#^, 2.1 *	(0.9954), 1.1 ^#^, 3.6 *	(0.9997), 0.3 ^#^, 0.9 *	(0.9907), 1.5 ^#^, 2.9 *
**6**	Y = 6.7935x + 0.0046	Y = 5.6243x − 0.0925	Y = 5.5703x + 0.016	Y = 7.3807x − 0.9386	Y = 5.5648x + 3.842	Y = 7.3829x − 1.2771
	(0.9990), 0.4 ^#^, 1.2 *	(0.9910), 0.5 ^#^, 1.5 *	(0.9978), 0.5 ^#^, 1.6 *	(0.9991), 0.5 ^#^, 1.6 *	(0.9978), 0.4 ^#^, 1.2 *	(0.9980), 0.7 ^#^, 2.3 *
**7**	Y = 1.1315x − 0.0233	Y = 0.9576x + 0.9475	Y = 0.8799x + 0.8954	Y = 1.2452x − 0.1016	Y = 0.9489x + 12.643	Y = 1.2494x − 0.2026
	(0.9970), 0.6 ^#^, 2.0 *	(0.9995), 0.9 ^#^, 3.1 *	(0.9961), 1.3 ^#^, 4.2 *	(0.9992), 0.9 ^#^, 3.0 *	(0.9998), 0.6 ^#^, 2.1 *	(0.9982), 0.7 ^#^, 2.2 *
**8**	Y = 14.335x − 0.7785	Y = 10.429x − 0.3695	Y = 17.239x − 3.9107	Y = 10.303x + 0.137	Y = 16.972x − 4.5354	Y = 12.86x − 2.3863
	(0.9999), 0.1 ^#^, 0.4 *	(0.9905), 0.2 ^#^, 3.1 *	(0.9966), 0.9 ^#^, 0.4 *	(0.9978), 0.7 ^#^, 2.5 *	(0.9955), 1.1 ^#^, 3.5 *	(0.9915), 1.1 ^#^, 3.7 *
**9**	Y = 6.8643x − 0.7974	Y = 5.629x + 0.0298	Y = 4.909x − 1.1473	Y = 6.0233x − 0.1356	Y = 5.7694x − 0.2297	Y = 7.4052x − 2.0624
	(0.9952), 0.8 ^#^, 2.8 *	(0.9919), 0.9 ^#^, 3.3 *	(0.9955), 1.1 ^#^, 3.1 *	(0.9969), 0.9 ^#^, 2.9 *	(0.9966), 0.9 ^#^, 3.0 *	(0.9941), 1.2 ^#^, 4.0 *
**10**	Y = 10.077x − 0.784	Y = 12.112x − 0.9891	Y = 11.679x − 2.3922	Y = 12.11x − 1.6105	Y = 11.615x − 2.5661	Y = 12.44x − 2.7397
	(0.9990), 0.4 ^#^, 1.2 *	(0.9900), 0.5 ^#^, 1.7 *	(0.9974), 0.8 ^#^, 2.7 *	(0.9989), 0.5 ^#^, 1.7 *	(0.9969), 0.9 ^#^, 3.0 *	(0.9964), 0.9 ^#^, 3.1 *

**Table 2:** Percentage recoveries, (percentage relative standard deviation (%RSD)) Intra-day ^+^, (%RSD) Inter-day *, and measurement uncertainty for the compounds in the solvent and different matrices.

	Compound 1	Compound 2	Compound 3	Compound 4	Compound 5	Compound 6	Compound 7	Compound 8	Compound 9	Compound 10
**Solvent**										
1 mg L^−1^	1.1 ^+^	0.9 ^+^	0.8 ^+^	0.4 ^+^	0.7 ^+^	0.8 ^+^	1.0 ^+^	0.3 ^+^	0.9 ^+^	0.8 ^+^
	1.6 *	1.0 *	0.3 *	0.4 *	1.1 *	0.4 *	0.6 *	1.1 *	1.6 *	0.2 *
3 mg L^−1^	0.4 ^+^	0.7 ^+^	0.6 ^+^	0.4 ^+^	0.4 ^+^	0.6 ^+^	0.4 ^+^	0.8 ^+^	0.2 ^+^	0.5 ^+^
	0.5 *	0.05 *	0.3 *	0.05 *	0.3 *	0.1 *	0.6 *	0.05 *	0.08 *	0.1 *
10 mg L^−1^	0.3 ^+^	0.2 ^+^	0.3 ^+^	0.4 ^+^	0.2 ^+^	0.2 ^+^	0.3 ^+^	0.2 ^+^	0.2 ^+^	0.2 ^+^
	0.02 *	0.03 *	0.1 *	0.03 *	0.1 *	0.05 *	0.2 *	0.05 *	0.2 *	0.05 *
**Leaves**										
3 mg kg^−1^	76.0	77.4	99.8	96.5	113.0	100.0	75.7	93.9	94.0	101.4
	4.3 ^+^	3.2 ^+^	0.4 ^+^	1.6 ^+^	0.9 ^+^	1.6 ^+^	1.8 ^+^	1.5 ^+^	1.9 ^+^	0.8 ^+^
	6.3 *	3.5 *	1.5 *	4.2 *	2.8 *	3.2 *	4.0 *	3.0 *	3.5 *	1.6 *
6 mg kg^−1^	89.5	91.6	100.5	93.7	104.7	99.6	84.8	100.3	100.0	100.4
	1.8 ^+^	0.8 ^+^	0.6 ^+^	1.3 ^+^	1.0 ^+^	1.7 ^+^	1.6 ^+^	0.9 ^+^	1.5 ^+^	0.4 ^+^
	2.5 *	2.3 *	1.2 *	0.9 *	0.6 *	0.3 *	1.8 *	0.2 *	0.4 *	0.4 *
10 mg kg^−1^	99.1	95.6	100.5	98.2	97.3	100.5	91.1	99.9	99.9	99.5
	0.4 ^+^	0.6 ^+^	0.4 ^+^	1.2 ^+^	0.3 ^+^	0.4 ^+^	0.5 ^+^	0.3 ^+^	0.6 ^+^	0.4 ^+^
	0.3 *	0.4 *	0.6 *	0.6 *	0.5 *	0.2 *	0.6 *	0.6 *	0.2 *	0.2 *
U (%)	8.1	7.4	0.3	1.2	4.0	0.4	7.0	2.1	1.6	0.5
**Porridge**										
3 mg kg^−1^	75.9	98.5	94.3	93.7	100.6	99.2	75.8	94.00	82.00	94.0
	2.3 ^+^	0.6 ^+^	0.5 ^+^	1.9 ^+^	1.2 ^+^	0.5 ^+^	1.6 ^+^	0.8 ^+^	1.7 ^+^	0.2 ^+^
	4.0 *	2.5 *	0.9 *	2.8 *	2.9 *	0.7 *	4.6 *	0.3 *	1.5 *	1.4 *
6 mg kg^−1^	87.5	101.6	97.0	96.4 *	101.0	100.8	90.9	96.00	91.33	96.8
	0.4 ^+^	0.5 ^+^	0.6 ^+^	1.3 ^+^	0.2 ^+^	0.5 ^+^	1.0 ^+^	0.2 ^+^	0.3 ^+^	0.5 ^+^
	1.6 *	1.0 *	1.0 *	3.6	4.7 *	1.2 *	4.9 *	1.9 *	0.2 *	0.9 *
10 mg kg^−1^	99.4	99.6	100.6	99.9	99.6	99.8	98.9	102.00	99.40	101.7
	0.7 ^+^	0.6 ^+^	0.2 ^+^	1.2 ^+^	0.3 ^+^	0.3 ^+^	1.0 ^+^	0.6 ^+^	0.7 ^+^	0.2 ^+^
	0.3 *	0.5 *	0.2 *	4.6 *	1.2 *	0.2 *	2.6 *	0.3 *	0.3 *	0.4
U (%)	7.3	2.9	1.9	5.7	3.2	3.2	7.5	1.9	5.4	2.3
**Pill**										
3 mg kg^−1^	72.0	99.3	97.7	78.0	82.00	94.3	99.3	100.2	99.3	87.3
	1.7 ^+^	0.5 ^+^	1.4 ^+^	2.0 ^+^	2.9 ^+^	1.0 ^+^	1.6 ^+^	0.8 ^+^	0.6 ^+^	1.4 ^+^
	4.4 *	1.4 *	0.5 *	1.7 *	1.0 *	0.3 *	3.0 *	2.4 *	0.3 *	0.4 *
6 mg kg^−1^	93.0	99.8	101.3	90.5	91.33	99.7	97.3	100.0	99.7	98.0
	1.1 ^+^	0.8 ^+^	0.6 ^+^	1.8 ^+^	0.4 ^+^	1.2 ^+^	1.4 ^+^	0.4 ^+^	0.6 ^+^	0.8 ^+^
	0.2 *	0.2 *	0.5 *	0.6 *	0.1 *	0.3 *	1.8 *	1.6 *	2.2 *	0.7 *
10 mg kg^−1^	98.2	100.1	99.7	99.1	99.40	100.6	101.0	99.8	100.3	101.1
	0.1 ^+^	0.8 ^+^	0.3 ^+^	1.0 ^+^	0.4 ^+^	0.6 ^+^	1.4 ^+^	0.3 ^+^	0.5 ^+^	0.2 ^+^
	0.4 *	0.6 *	0.4 *	0.3 *	0.2 *	0.2 *	0.5 *	0.2 *	0.2 *	0.7 *
U (%)	9.3	1.5	0.9	6.6	6.0	1.7	0.5	2.4	0.4	3.7
**Seeds**										
3 mg kg^−1^	66.7	95.3	93.7	96.0	97.0	100.0	108.9	92.3	98.4	93.7
	4.7 ^+^	0.6 ^+^	0.8 ^+^	1.8 ^+^	1.0 ^+^	0.4 ^+^	0.7 ^+^	0.6 ^+^	0.4 ^+^	1.3 ^+^
	6.0 *	0.6 *	0.8 *	4.1 *	2.4 *	0.5 *	0.7 *	2.8 *	0.6 *	1.4 *
6 mg kg^−1^	84.2	96.5	96.7	100.8	101.3	100.6	105.4	95.5	99.7	96.3
	0.3 ^+^	0.2 ^+^	0.4 ^+^	1.7 ^+^	0.3 ^+^	0.2 ^+^	0.6 ^+^	0.2 ^+^	0.4 ^+^	0.3 ^+^
	0.2 *	0.5 *	0.6 *	4.4 *	0.5 *	0.8 *	0.2 *	0.5 *	0.5 *	0.3 *
10 mg kg^−1^	99.0	101.7	101.8	100.0	99.7	99.9	100.1	102.3	100.2	101.9
	0.2 ^+^	0.3 ^+^	0.3 ^+^	1.4 ^+^	0.3 ^+^	0.3 ^+^	0.3 ^+^	0.1 ^+^	0.3 ^+^	0.2 ^+^
	0.2 *	0.3 *	1.2 *	3.5 *	1.3 *	0.4 *	0.8 *	0.2 *	1.2 *	0.5 *
U (%)	10.7	1.6	1.7	5.5	2.8	0.6	2.8	3.6	1.4	2.4
**Teabags**										
3 mg kg^−1^	86.2	69.7	93.7	99.2	96.3	95.3	96.0	98.9	93.7	94.7
	4.3 ^+^	4.5 ^+^	2.0 ^+^	2.0 ^+^	1.3 ^+^	1.4 ^+^	1.9 ^+^	1.5 ^+^	1.9 ^+^	1.2 ^+^
	6.3 *	5.7 *	1.1 *	3.5 *	2.6 *	2.7 *	2.0 *	1.2 *	3.6 *	3.7 *
6 mg kg^−1^	94.1	91.0	95.7	97.8	91.2	97.0	96.8	93.2	93.8	95.3
	3.2 ^+^	3.1 ^+^	0.5 ^+^	0.5 ^+^	0.5 ^+^	0.9 ^+^	1.3 ^+^	0.3 ^+^	0.3 ^+^	1.2 ^+^
	3.2 *	3.5 *	0.5 *	3.3 *	0.6 *	0.7 *	3.7 *	1.2 *	0.5 *	1.6 *
10 mg kg^−1^	103.6	98.2	102.3	100.3	103.6	101.5	101.5	102.6	102.8	102.1
	0.4 ^+^	0.1 ^+^	0.6 ^+^	0.6 ^+^	0.7 ^+^	0.1 ^+^	1.3 ^+^	0.2 ^+^	0.3 ^+^	0.4 ^+^
	0.6 *	0.5 *	0.5 *	2.9 *	2.3 *	1.4 *	3.1 *	0.3 *	0.8 *	0.9 *
U (%)	6.0	9.8	2.3	4.6	3.8	3.3	3.9	1.5	4.1	4.1

**Table 3:** Quantification data for application of the validated method to real samples (reported in mg kg^−1^ of the compound per 280 mg kg^−1^ of the sample).

	Region	Compound 1	Compound 2	Compound 3	Compound 4	Compound 5	Compound 6	Compound 7	Compound 8	Compound 9	Compound 10
**L1**	Mpumalanga (SA)	0.0	0.1	6.9	0.1	0.1	6.9	7.4	9.4	0.00	5.6
**L2**	Mpumalanga (SA)	0.0	0.0	6.1	0.0	0.1	8.7	7.0	7.0	0.0	7.9
**L3**	Mpumalanga (SA)	0.0	0.0	5.9	0.0	0.1	9.7	7.5	7.0	0.0	6.5
**L4**	Mpumalanga (SA)	0.1	0.1	8.4	0.1	0.1	5.0	7.5	8.6	0.1	6.6
**L5**	Limpopo (SA)	0.0	0.1	5.0	0.0	0.0	6.5	6.4	0.0	0.0	5.9
**L6**	Limpopo (SA)	0.1	0.1	5.0	0.0	0.1	8.8	5.0	0.1	0.0	6.9
**L7**	Limpopo (SA)	0.0	0.1	6.4	0.00	0.0	5.2	8.9	0.0	0.0	7.4
**L8**	Limpopo (SA)	0.0	0.1	6.1	0.0	5.4	7.0	8.0	0.0	0.0	7.1
**L9**	Ethiopia	0.0	0.0	5.8	0.0	0.1	4.2	7.1	0.1	0.0	0.1
**P1**	MI (SA)	0.0	0.1	5.6	0.0	0.0	4.5	6.6	0.1	0.0	5.7
**P2**	MI (SA)	0.0	0.1	5.2	0.0	0.1	5.1	6.9	0.1	0.0	6.0
**P3**	MII (SA)	0.0	0.1	6.9	0.0	5.0	6.2	8.6	0.0	0.0	7.7
**P4**	MII (SA)	0.1	0.1	5.7	0.0	0.1	6.7	0.1	0.1	0.0	7.6
**P5**	MIII (SA)	0.1	6.0	0.0	0.0	5.9	5.0	7.3	0.1	0.0	5.8
**P6**	MIII (SA)	0.1	5.7	0.0	0.0	5.6	6.1	8.5	0.1	0.0	5.2
**PRD 1**	MI (SA)	0.1	0.0	6.0	0.1	5.8	6.0	9.6	4.5	0.0	0.1
**PRD 2**	MI (SA)	0.1	0.0	5.9	0.1	6.0	6.0	9.1	4.5	0.0	0.1
**PRD3**	MIV (SA)	6.9	0.0	8.0	5.1	6.9	6.8	0.1	0.0	0.0	9.3
**PRD4**	MIV (SA)	5.3	0.0	7.6	5.0	6.5	5.8	0.1	0.0	0.0	8.8
**TB1**	MI (SA)	0.0	0.1	6.6	0.0	0.0	7.0	7.2	5.8	0.0	0.0
**TB2**	MI (SA)	0.1	0.0	6.4	0.0	0.0	4.2	6.5	4.6	0.0	0.0
**TB3**	MII (SA)	0.0	0.0	8.2	5.0	0.0	6.1	8.0	6.2	0.0	0.0
**TB4**	MII (SA)	0.1	0.0	8.1	6.8	0.0	7.0	8.1	5.9	0.0	0.0
**TB5**	MIV (SA)	0.0	5.6	5.0	0.0	0.0	4.1	6.9	4.2	0.0	0.1
**TB6**	MIV (SA)	0.0	6.2	6.0	0.0	0.0	4.2	6.6	4.6	0.0	0.1
**TB7**	Jamaica	6.5	0.0	6.3	0.1	8.7	8.5	7.0	4.7	0.0	6.1
**S1**	Mpumalanga (SA)	6.4	4.7	0.0	0.0	0.1	6.5	9.6	0.0	4.7	0.0
**S2**	Mpumalanga (SA)	5.2	0.1	0.1	0.0	0.1	7.7	8.8	0.0	0.1	0.1
**S3**	Mpumalanga (SA)	5.6	0.1	0.1	0.0	0.1	5.1	8.1	0.0	0.1	0.0
**S4**	Limpopo (SA)	5.0	4.7	0.1	0.0	0.1	5.5	9.5	0.0	4.9	0.1
**S5**	Limpopo (SA)	9.4	4.6	0.0	0.0	0.1	5.1	6.7	0.0	0.0	0.1
**S6**	Zambia	0.0	5.8	0.1	0.0	6.9	5.4	0.1	0.0	5.6	0.0

L = Leaves; PRD = Porridge; TB = Teabag; 0.1 mg kg^−1^ = Not quantified; P = Pill; S = Seeds; 0.0 mg kg^−1^ = Not detected; M = Manufacturer.

## Supplementary Materials

The following are available online. Figure S1: Composition of mild and medium matrix effect in the different products, Figure S2: Hierarchical clustering of the samples, Figure S3: A chromatogram representing the standards in the solvent and their UV spectra, Figures S4–S8: Chromatograms representing samples L**4**, P**4**, PRD**1**, S**2**, and TB**7** respectively, and the UV spectra of the compounds of interest, Table S1: Information about reference standards used to develop a separation and quantification method, Table S2: Samples information.
