# Supplementary material for: Development of an HPLC-DAD Method for the Quantification of Ten Compounds from Moringa oleifera Lam. and Its Application in Quality Control of Commercial Products
Source: Molecules. 2020 Sep 28;25(19):4451. doi: 10.3390/molecules25194451 (PMC7583788; doi:10.3390/molecules25194451)
Supplement: Supplementary file 1 [file molecules-25-04451-s001.pdf]

Table S1: Reference standards used to develop a separation and quantification method

Compound name	Compound number	CAS number	Molecular mass	Supplier
3,4,5-trihydroxybenzoic acid	1	149-91-7	170.12 g mol^{-1}	Sigma-Aldrich
3-caffeoylquinic acid	2	N/A	355.1019 g mol^{-1}	Sigma- Aldrich
4-hydroxy-3-methoxylbenzaldehyde	3	121-33-5	152.15 g mol^{-1}	Sigma-Aldrich
2,3,7,8-tetrahydroxychromen [5,4,3-cde]chromen-5,10-dion	4	476-66-4	302.19 g mol^{-1}	Sigma-Aldrich

(E)-3-(4-hydroxy-3-methoxyphenyl)prop-2-enoic acid	5	537-98-4	194.18 g mol^{-1}	Sigma-Aldrich
O-ethyl-[4-(α-L-rhamnosyloxy) benzyl] thiocarbamate	6		358.1401 g mol^{-1}	Isolated
(4-α-L-rhamnosyloxy)-benzyl isothiocyanate	7	N/A	334.0797 g mol^{-1}	Isolated
2-(3,4-dihydroxyphenyl)-3,5,7-trihydroxychromen-4-one	8	117-39-5	302.24 g mol^{-1}	Sigma-Aldrich
O-butyl-[4-(α-L-rhamnosyloxy) benzyl] thiocarbamate	9	N/A	386.1719 g mol^{-1}	Isolated

3,5,7-trihydroxy-2-(4-hydroxyphenyl)chromen-4-one	10	520-18-3	286.24 g mol^{-1}	Sigma-Aldrich

3

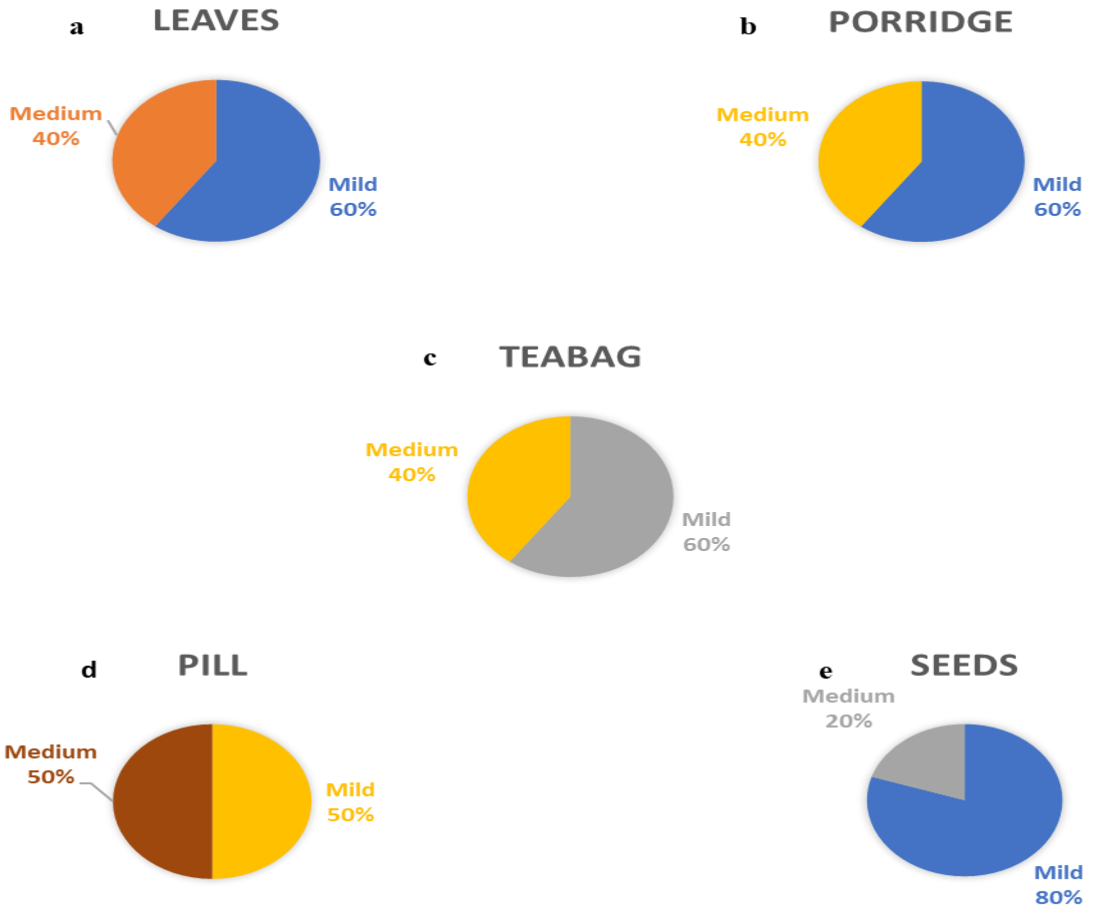

Figure S1: Composition of mild and medium matrix effect in the different products

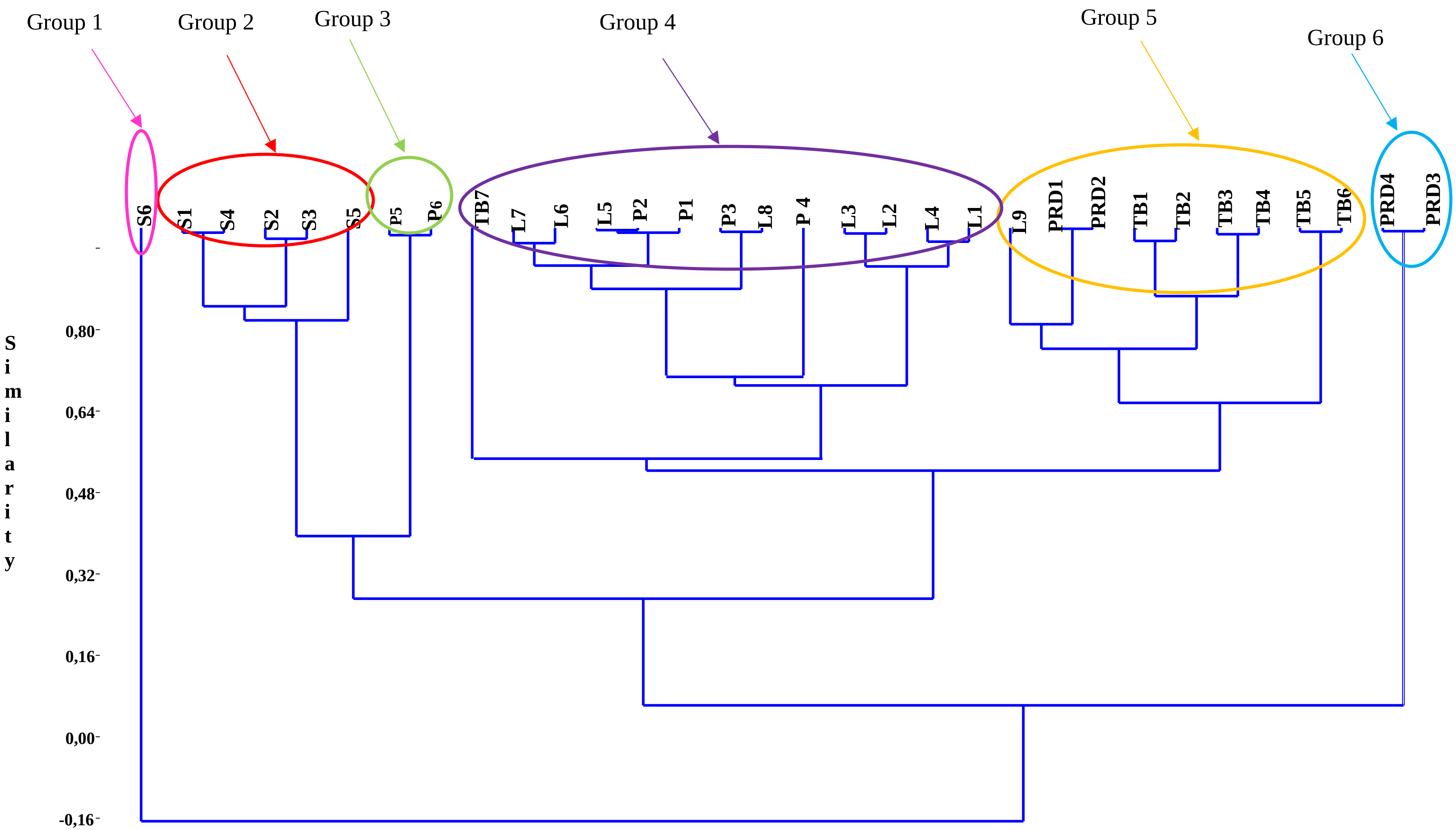

Figure S2: Hierarchical clustering of the samples

Table S2: Samples information

**Sample**	**Country**	**Region/ company name**	**GPS coordinates**	**Month collected**	**Voucher species#**
**L1**	South Africa	Mpumalanga	25.1112°S, 28.5583°E	April	267
**L2**	South Africa	Mpumalanga	19.6633°S, 22.1937°E	April	268
**L3**	South Africa	Mpumalanga	25.0104°S, 28.8014°E	April	269
**L4**	South Africa	Mpumalanga	25.0259°S, 28.7023°E	May	270
**L5**	South Africa	Limpopo	23.8962°S, 29.4486°E	May	271
**L6**	South Africa	Limpopo	24.3110°S, 29.4753°E	May	272
**L7**	South Africa	Limpopo	24.8844°S, 28.3287°E	May	273
**L8**	South Africa	Limpopo	23.8477°S, 29.3933°E	May	274
**L9**	Ethiopia	Arbaminch	6.0205°N, 37.5641°e	Unknown	275
**S1**	South Africa	Mpumalanga	25.1112°S, 28.5583°E	April	276
**S2**	South Africa	Mpumalanga	19.6633°S, 22.1937°E	April	278
**S3**	South Africa	Mpumalanga	25.0104°S, 28.8014°E	April	279
**S4**	South Africa	Limpopo	23.8477°S, 29.3933°E	May	280
**S5**	South Africa	Limpopo	24.3110°S, 29.4753°E	May	281
**S6**	Zambia	Unknown	Unknown	Unknown	282

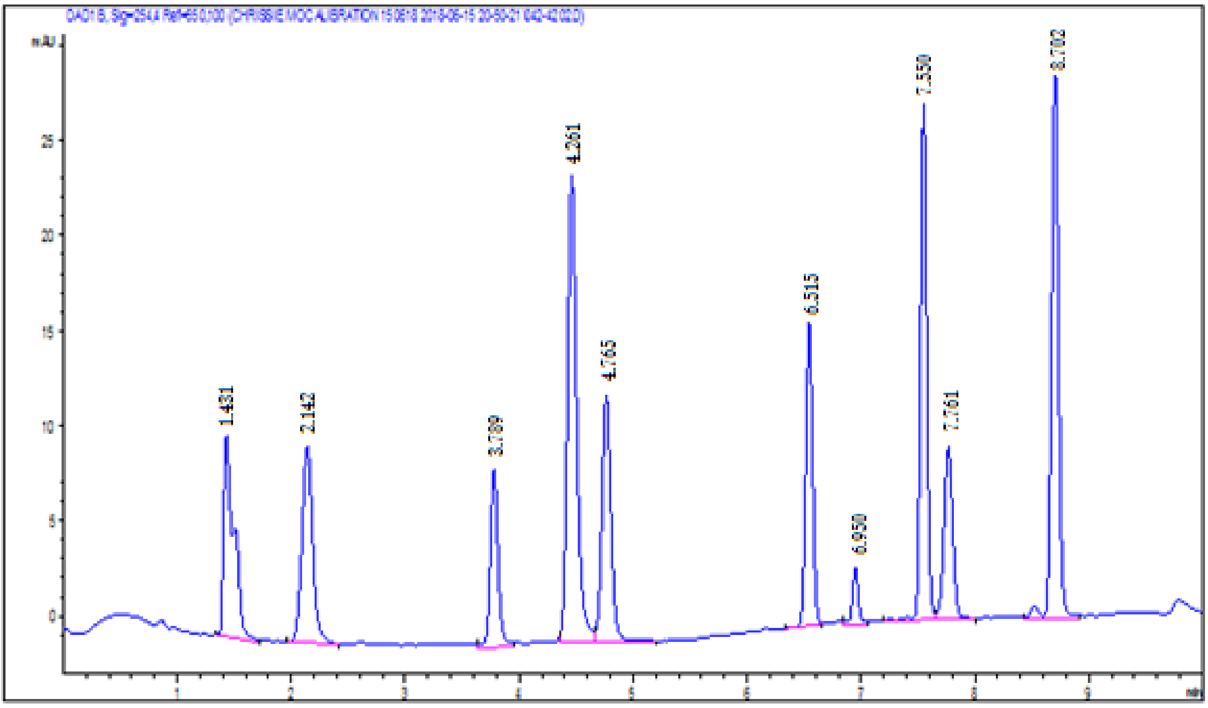

Figure S3: Chromatogram for the standards at 10 mg/L in the solvent

UV- Vis Spectra for the compound 1 – 10 in the solvent

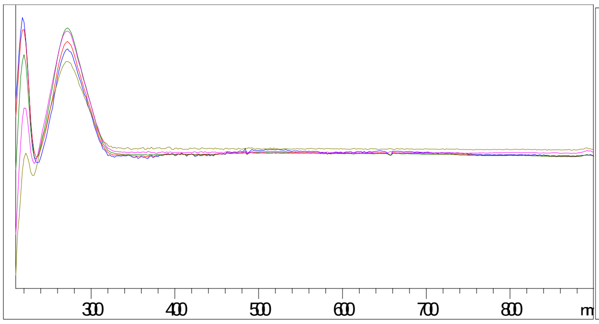

Compound 1

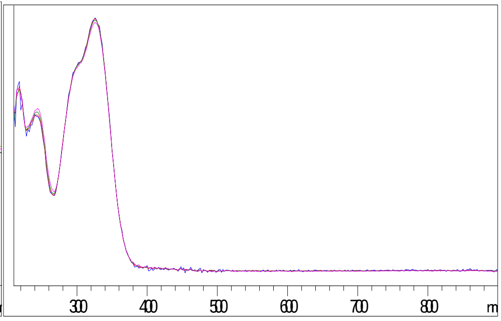

Compound 2

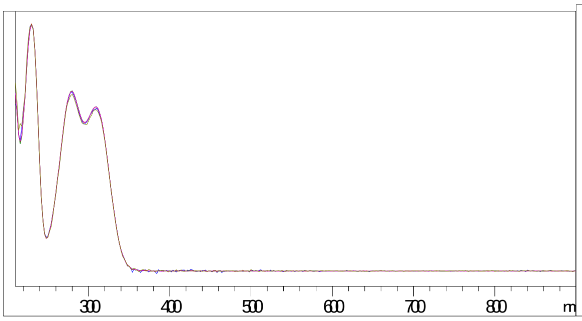

Compound 3

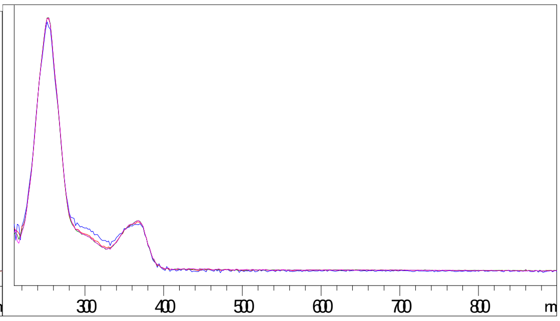

Compound 4

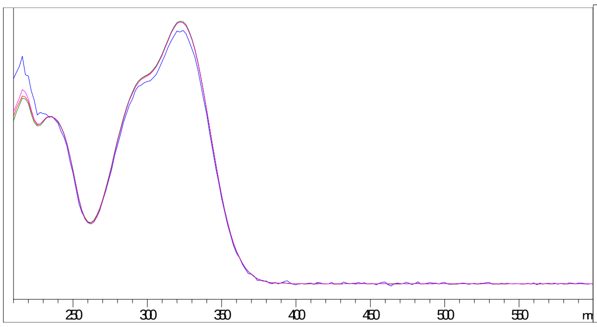

Compound 5

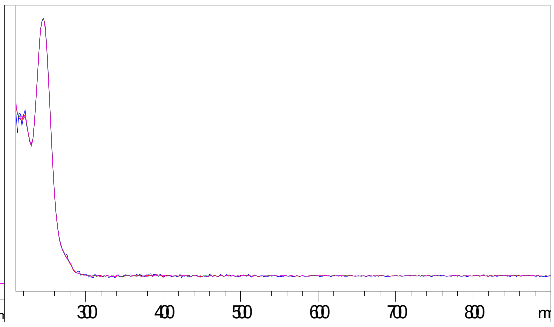

Compound 6

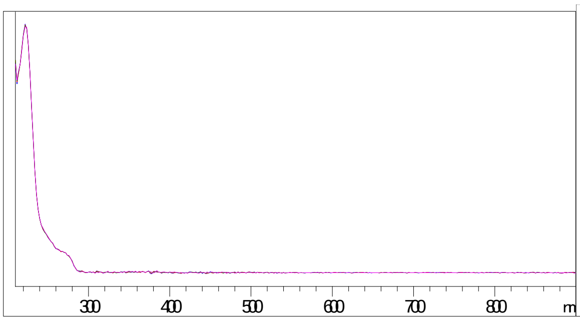

Compound 7

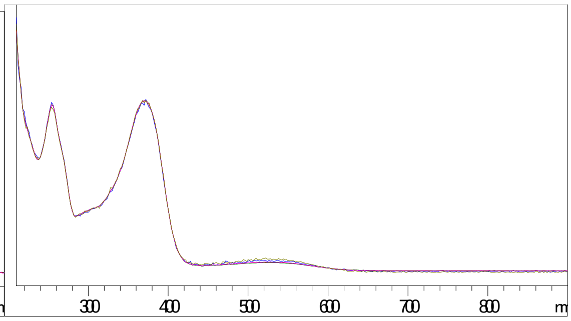

Compound 8

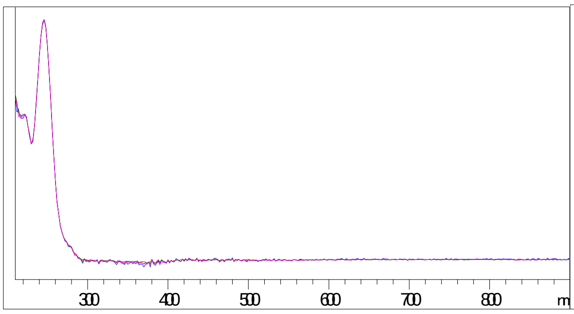

Compound 9

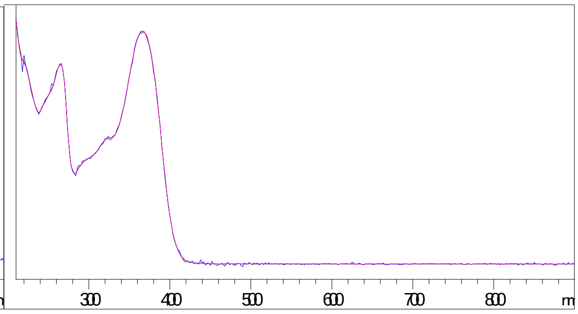

Compound 10

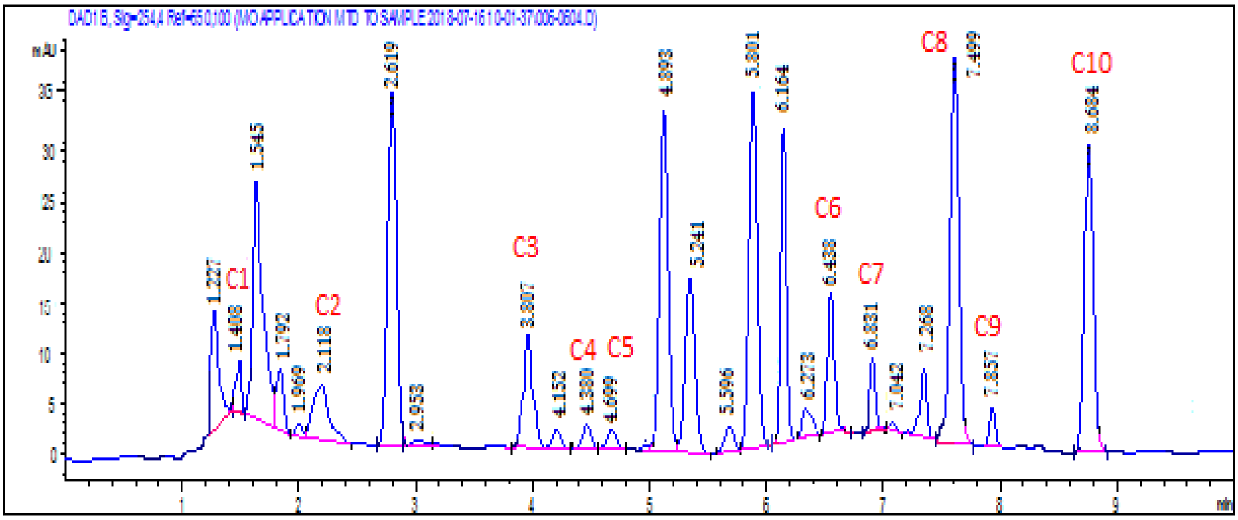

Figure S4: Chromatogram representing L4 separated using the validated method

UV- Vis Spectra of the compounds of interest identified in L4

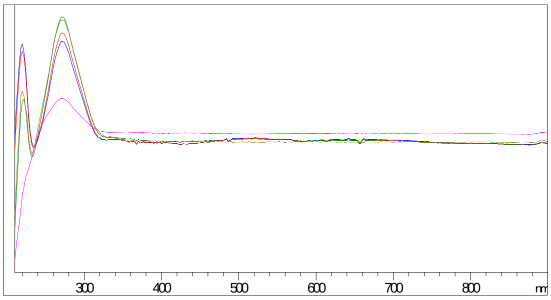

Compound 1

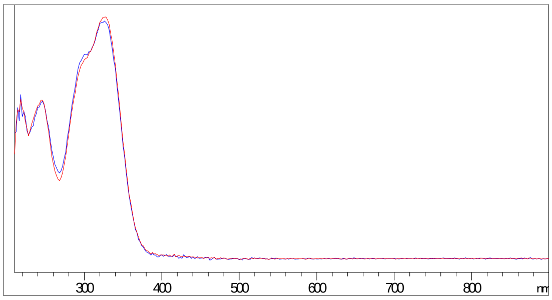

Compound 2

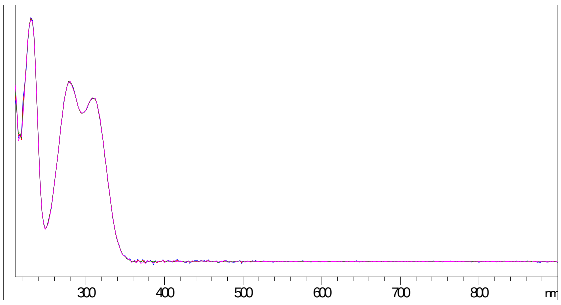

Compound 3

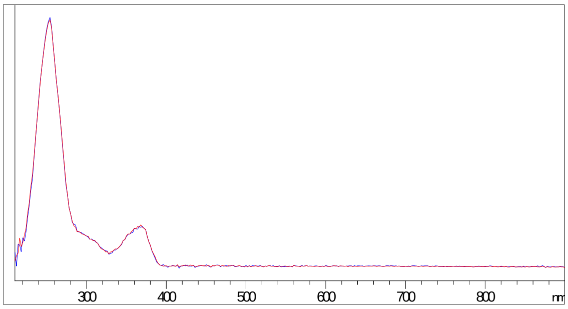

Compound 4

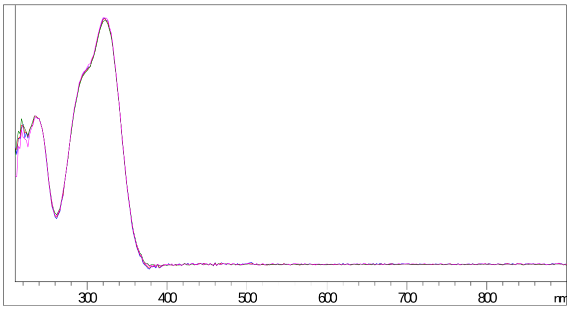

Compound 5

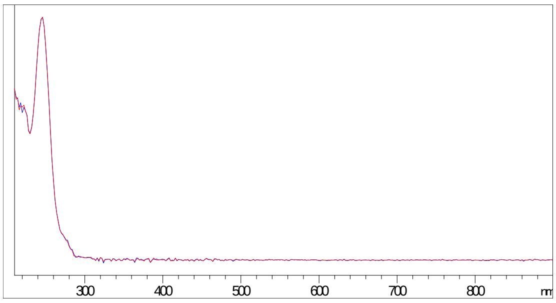

Compound 6

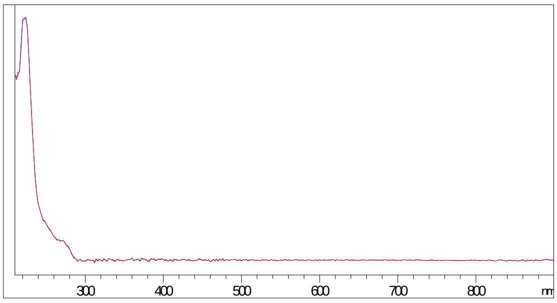

Compound 7

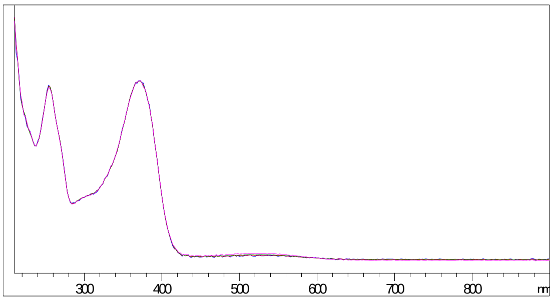

Compound 8

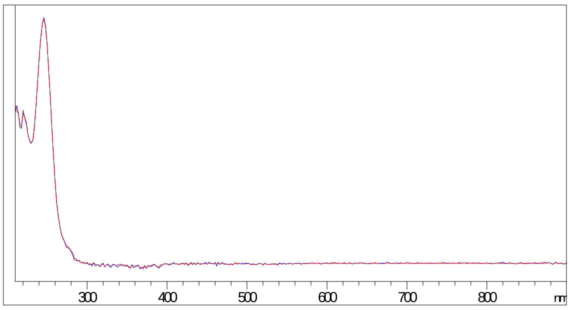

Compound 9

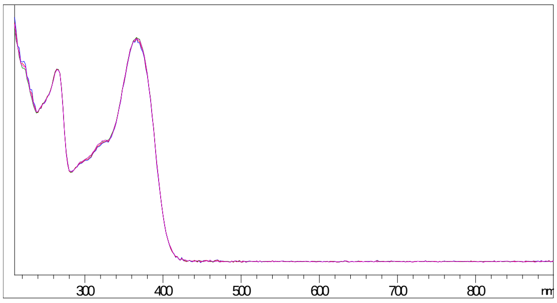

Compound 10

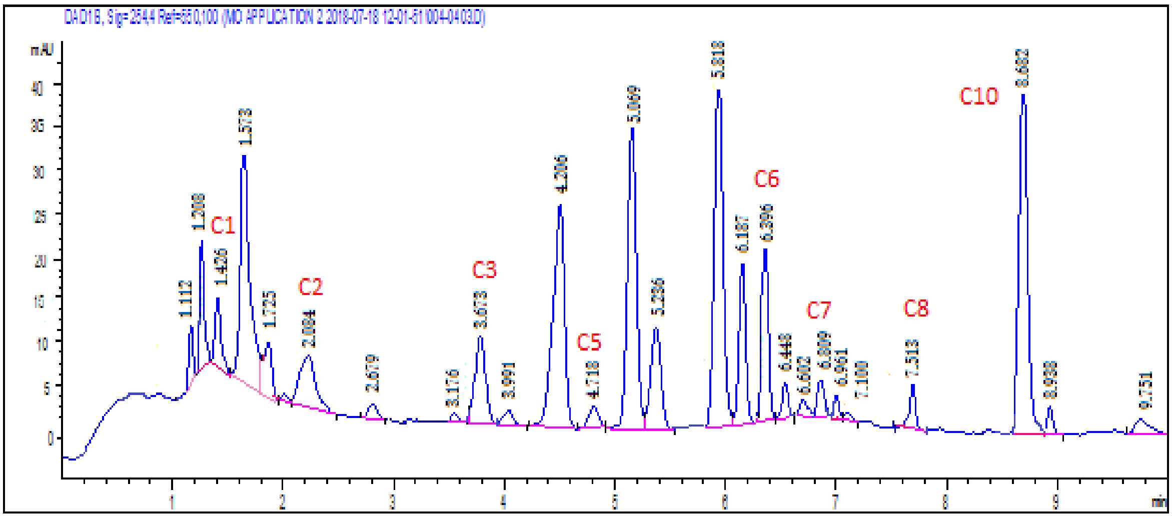

Figure S5: Chromatogram representing P4 separated using the validated method

UV- Vis Spectra of the compounds of interest identified in P4

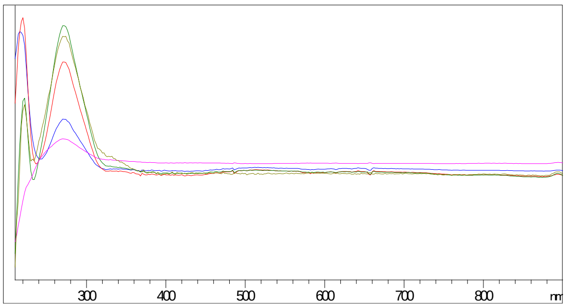

Compound 1

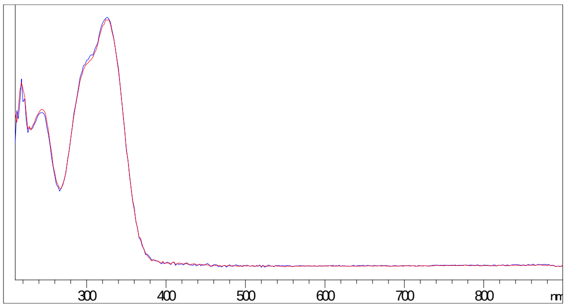

Compound 2

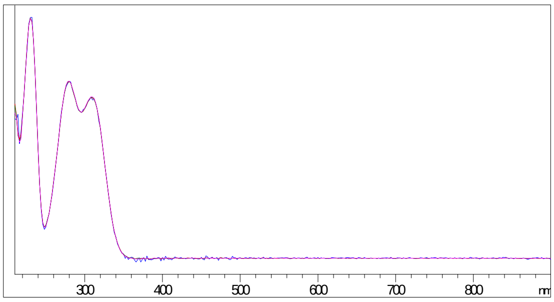

Compound 3

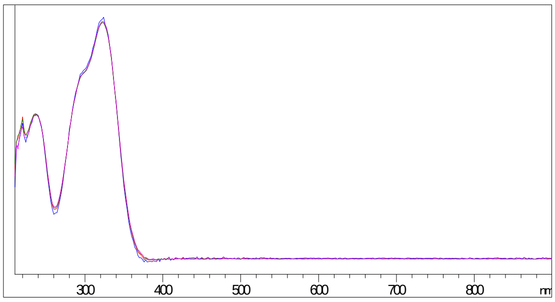

Compound 5

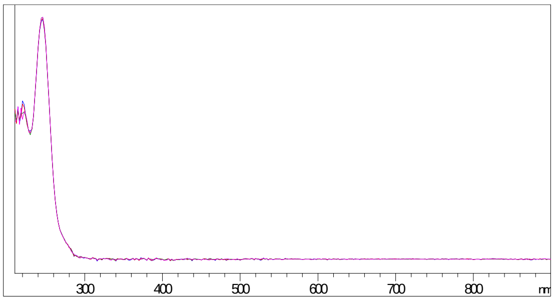

Compound 6

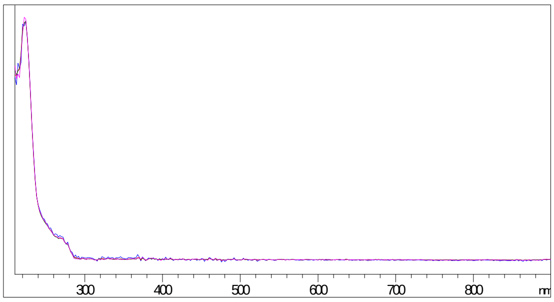

Compound 7

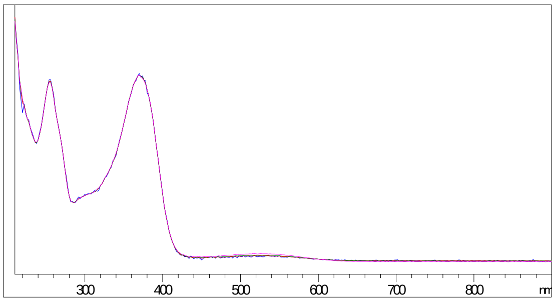

Compound 8

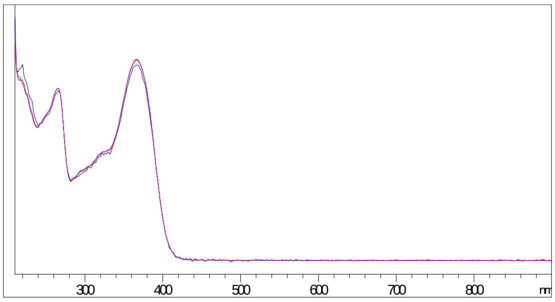

Compound 10

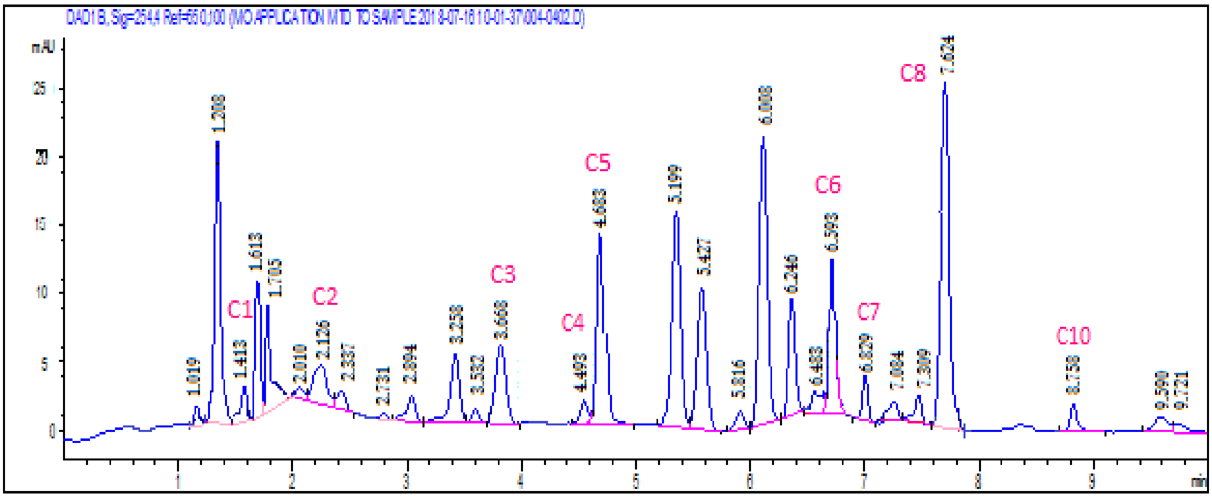

Figure S6: Chromatogram representing PRD1 separated using the validated method

UV-Vis Spectra of the compounds of interest identified in PRD1

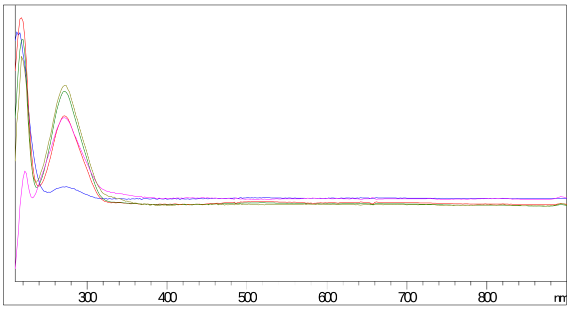

Compound 1

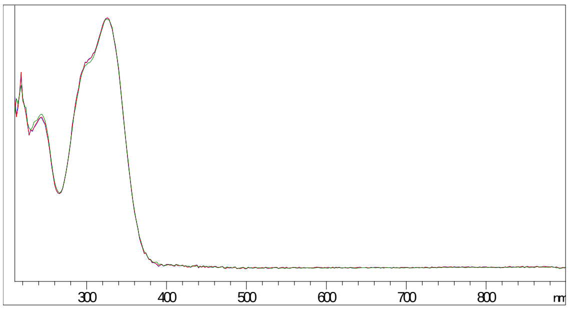

Compound 2

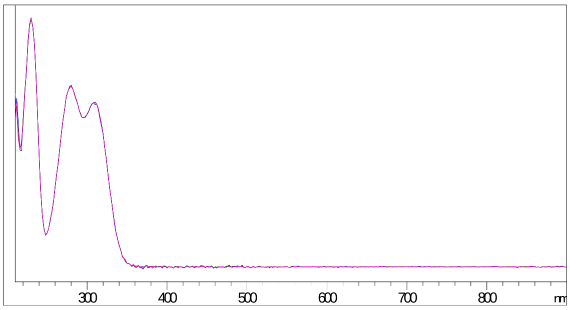

Compound 3

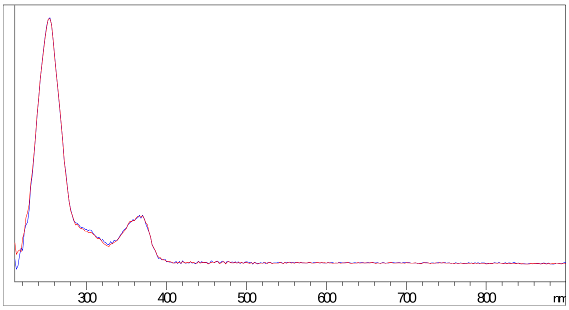

Compound 4

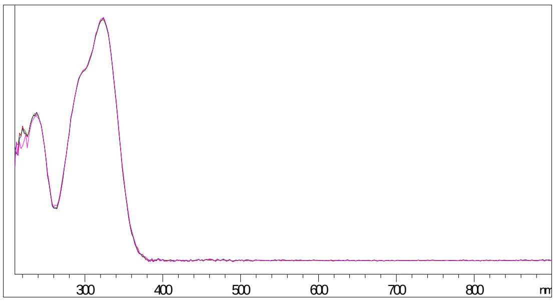

Compound 5

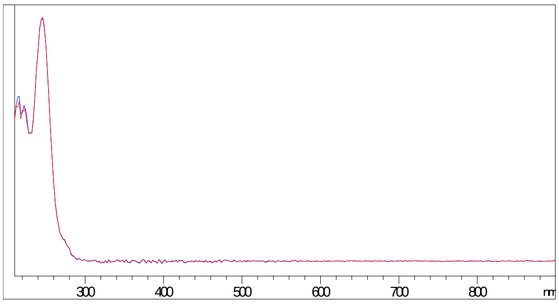

Compound 6

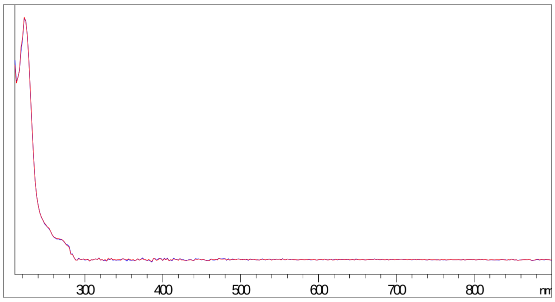

Compound 7

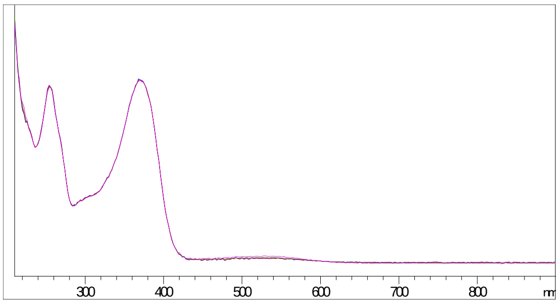

Compound 8

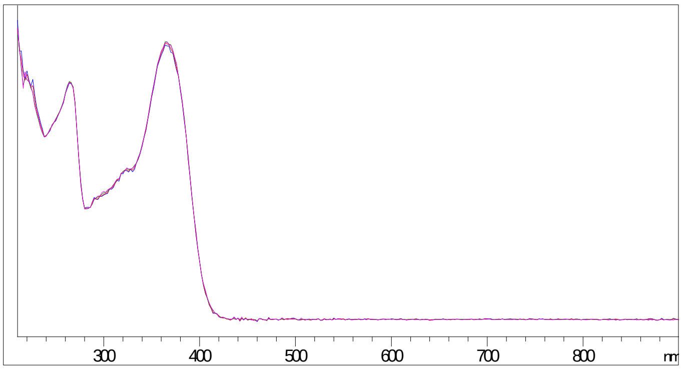

Compound 10

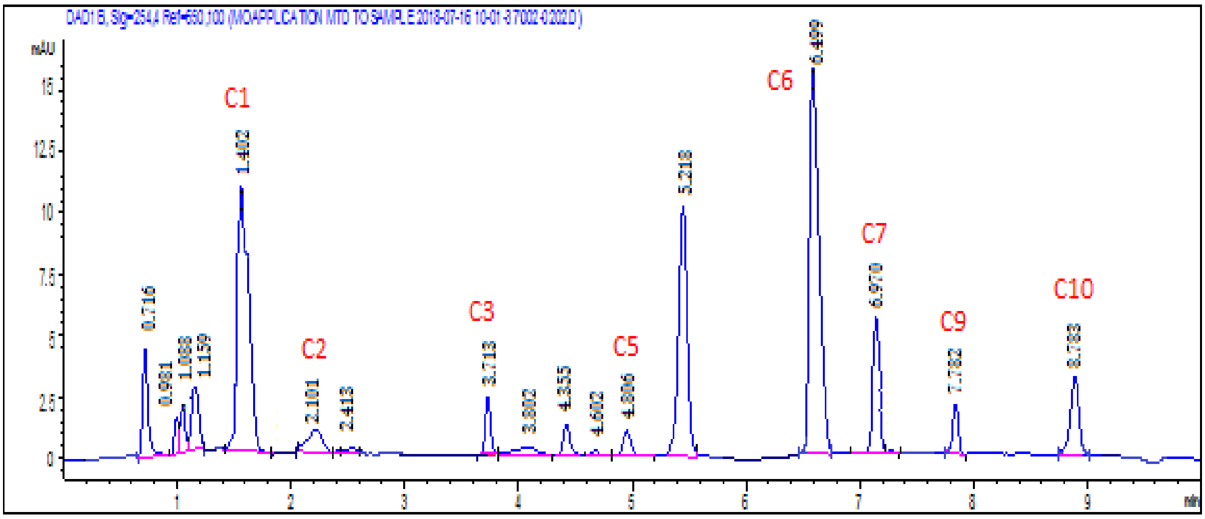

Figure S7: Chromatogram representing S2 separated using the validated method

UV- Vis Spectra of the compounds of interest identified in S2

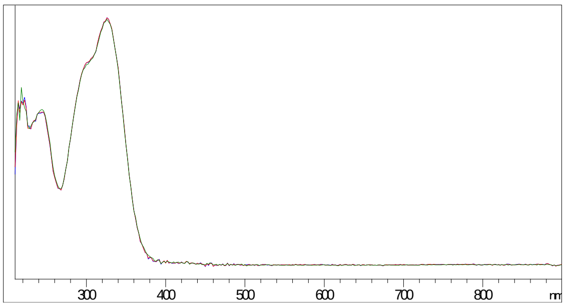

Compound 1

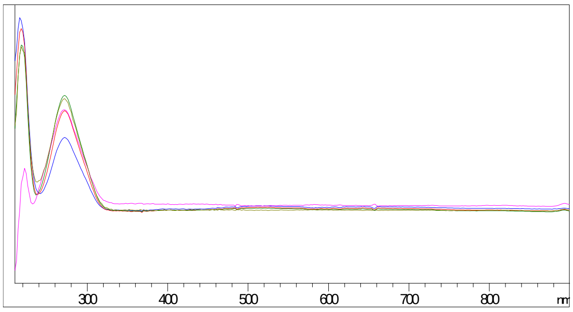

Compound 2

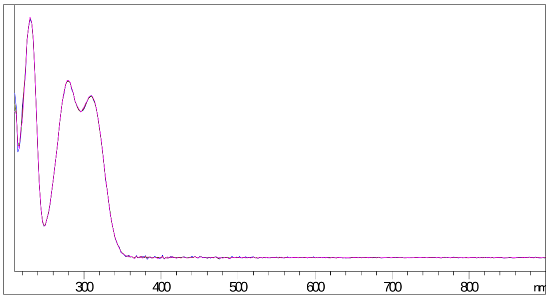

Compound 3

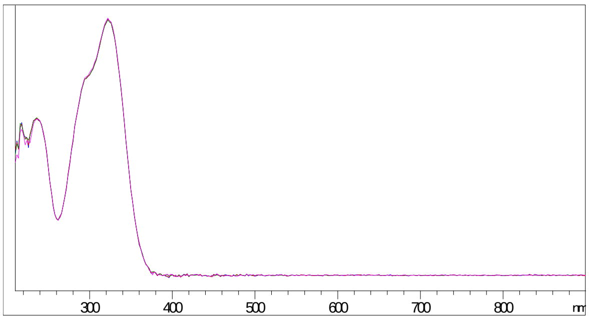

Compound 5

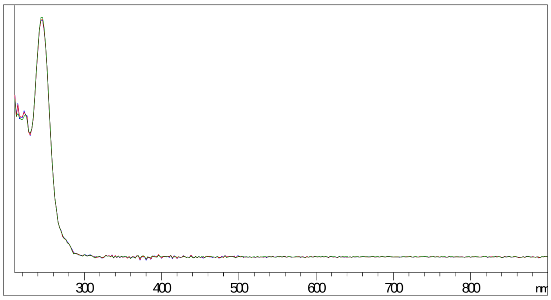

Compound 6

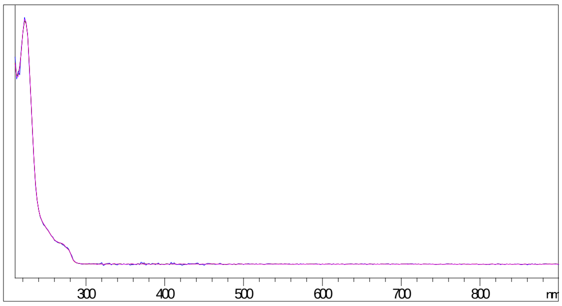

Compound 7

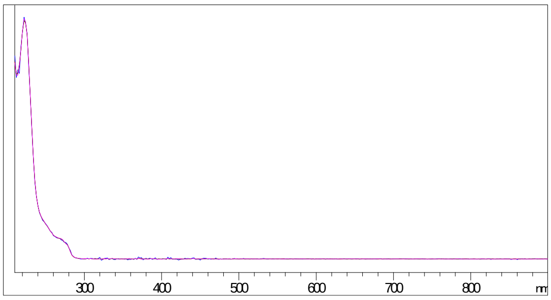

Compound 9

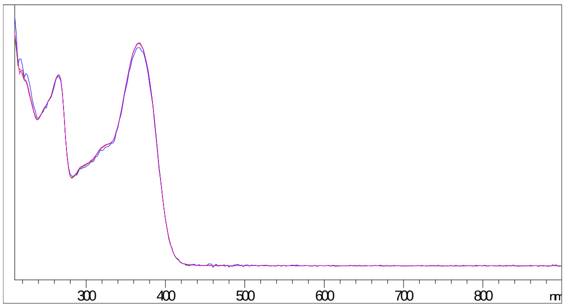

Compound 10

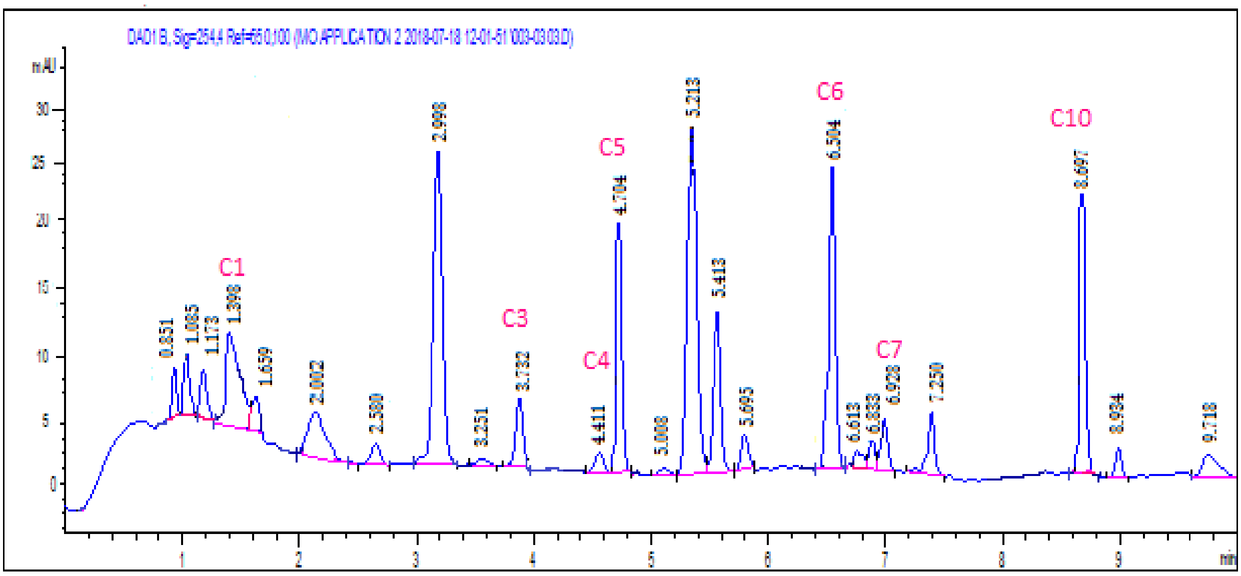

Figure S8: Chromatogram representing TB 7 separated using the validated method

UV- Vis Spectra of the compounds of interest identified in TB 7

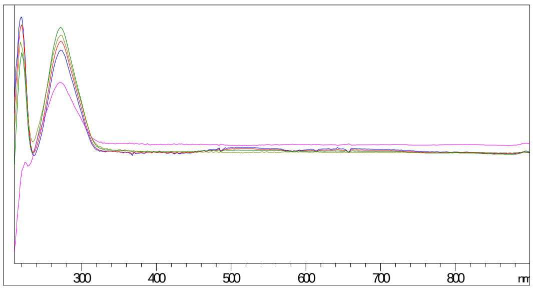

Compound 1

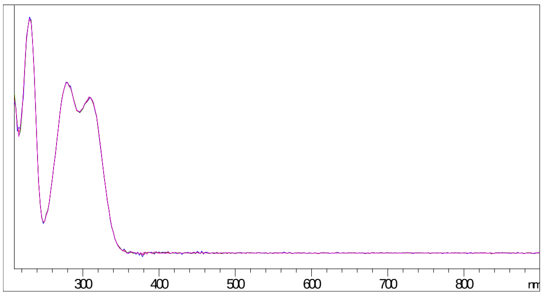

Compound 3

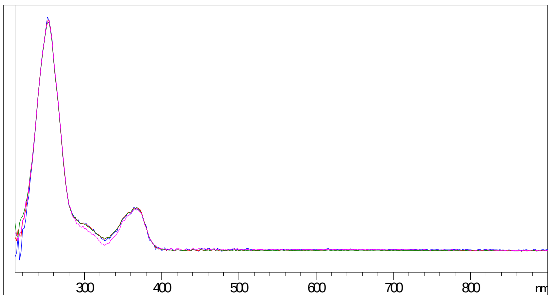

Compound 4

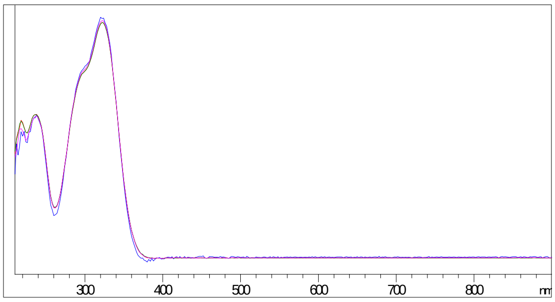

Compound 5

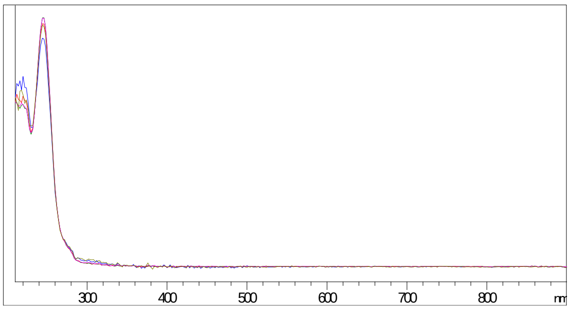

Compound 6

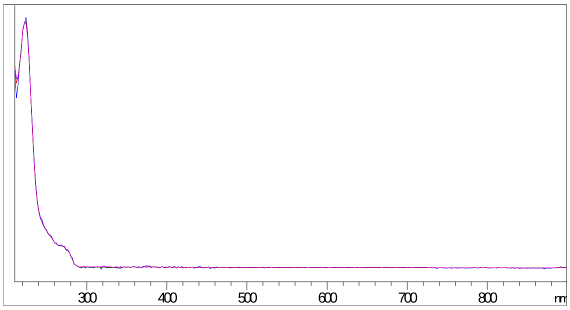

Compound 7

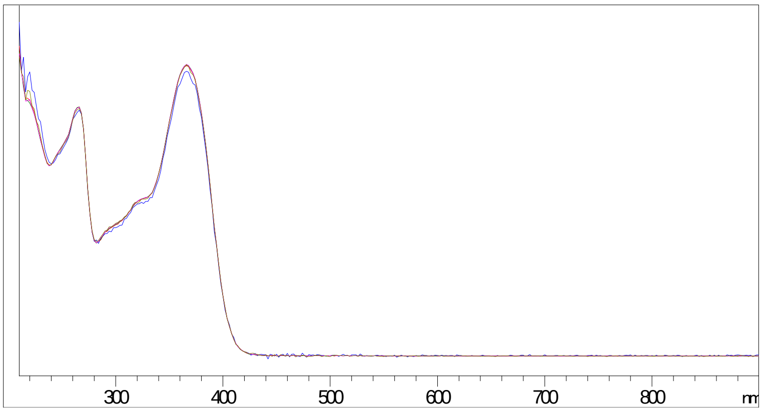

Compound 10
